# Recovery After an Official Soccer Match: An Analysis of Markers of Muscle Damage and Oxidative Stress, and Endocrine, Neuromuscular and Perceptual Responses

**DOI:** 10.3390/jfmk10030351

**Published:** 2025-09-13

**Authors:** Diego Marqués-Jiménez, Miguel Ramirez-Jimenez, José M. Izquierdo, José Losa-Reyna, Domingo Machuca Calvo, Jorge López-López, Daniel Castillo

**Affiliations:** 1 Valoración del Rendimiento Deportivo, Actividad Física y Salud y Lesiones Deportivas (REDAFLED), Department of Didactics of Musical, Plastic and Corporal Expression, Faculty of Education, University of Valladolid, 42004 Soria, Spain; diego.marques@uva.es (D.M.-J.); miguel.ramirez.jimenez@uva.es (M.R.-J.); josemaria.izquierdo@uva.es (J.M.I.); 2 Sports Science Research Centre, King Juan Carlos University, 28032 Fuenlabrada, Spain; jose.losa@urjc.es; 3 CIBER of Frailty and Healthy Aging (CIBERFES), 28029 Madrid, Spain; 4 Hospital Latorre, 42005 Soria, Spain; domingo.machuca@hospital-latorre.com (D.M.C.); jorgelopez81@gmail.com (J.L.-L.)

**Keywords:** biomarkers, fatigue, football, performance, wellness

## Abstract

**Objectives**: This study analysed the recovery process after an official soccer match by monitoring changes in markers of muscle damage and oxidative stress, and endocrine, neuromuscular, and perceptual responses. **Methods**: This repeated-measures observational study included thirteen male amateur soccer players. Blood biomarkers, neuromuscular performance in countermovement jump, and perceived wellness were measured at four time-points: the morning of the match-day, immediately post-, and 24 h and 48 h post-match. **Results**: Except for CK, which remained elevated at 48 h post-match, lactate dehydrogenase, C-reactive protein, uric acid, testosterone, cortisol, and testosterone to cortisol ratio returned to baseline between 24 h and 48 h post-match (*p* < 0.05). Jump height was significantly decreased at 24 h and 48 h post-match, while peak rate of force development and other countermovement jump time-based metrics (i.e., time to take off, time to peak force, reactive strength index modified, flight time to contraction time ratio) were impaired immediately after the match and recovered earlier (*p* < 0.05). Peak values for perceived fatigue and delayed onset muscle soreness were observed immediately post- and at 24 h post-match, respectively (*p* < 0.05). **Conclusions**: While certain physiological, neuromuscular, and perceptual changes may return to baseline levels within 24 h or 48 h post-match, amateur soccer players still manifest exercise-induced muscle damage symptoms and can be considered fatigued after a 48 h recovery period.

## 1. Introduction

The physiological and psychological demands during training and competition can decrease an athlete’s performance due to fatigue [1]. Fatigue is a reduced capacity to produce voluntary force, which typically lasts longer than the exercise itself and manifests as impaired muscle function and/or a reduced capacity of the central nervous system to activate muscles [2]. The nature of fatigue is task-dependent [3], and its magnitude relies on several characteristics of the exercise stimulus (e.g., type, volume, intensity) and the extent of stress on various physiological systems (e.g., metabolic, neuromuscular, cardiocirculatory), but also on individual characteristics (e.g., training status, habitual physical activity, sleep, psychological stress, genetic influences) [1,4]. Fatigue can be compensated with recovery, a multifaceted process that restores physiological and psychological resources and reestablishes the body’s balance after a temporary disturbance [5]. As such, fatigue and recovery can be viewed as a continuum, jointly affected by physiological and psychological factors [5]. In fact, the time required to fully recover is proportional to the level of fatigue [1].

An adequate balance between physiological and psychological stress and recovery is essential for athletes to achieve continuous high-level performance [5]. For soccer players, analysing the recovery process is critical but complex for coaches, performance staff, and physicians due to its multifactorial nature. A soccer match is a stimulus that disrupts homeostasis, causing acute (i.e., during or immediately after a match) and residual fatigue that can persist for 48 h, 72 h, or even 96 h post-match [6,7,8]. Mechanical strain and subsequent exercise-induced muscle damage (EIMD) resulting from the match-play also produce structural disruptions of the contractile elements within muscle fibres [9]. The muscle remodelling involves an inflammatory response, the degradation of damaged proteins, the synthesis of new proteins, endocrine alterations, and other responses and interactions (e.g., growth factors and cytokines) [10]. The outcomes from such disturbances may be delayed onset muscle soreness (DOMS) or impaired physical performance for up to several days after matches [6,7,8]. Additionally, soccer competition exacerbates self-reported perceptual measures of wellness with peak values observed immediately post-match and after 24 h of recovery [6]. Thus, recovery from soccer matches involves an integrated response from several psychological and physiological systems (e.g., metabolic, neuromuscular, cardiocirculatory).

Despite significant efforts to summarise the physiological and psychological responses during the recovery period after a soccer match [6,7,8,11,12], limitations from study heterogeneity can compromise the information available for drawing robust conclusions. For instance, some studies were conducted on amateur or semi-professional players during friendly matches played in the offseason [9,13], while others with similar samples did not report the period when the friendly match was played [14]. In fact, only a few studies have been conducted with amateur or semi-professional soccer players during the competitive period or mid-season [15,16,17], and only one was carried out during an official competition [16]. Given that official matches represent the most sport-specific competitive situations, further research on this population in these scenarios could provide interesting insights into players’ physiological and psychological recovery, help guide training schedules or recovery protocols, and improve the applicability to real-world conditions. Thus, this study analysed the recovery process after an official soccer match by monitoring changes in markers of muscle damage and oxidative stress, and endocrine, neuromuscular, and perceptual responses.

## 2. Materials and Methods

### 2.1. Participants

Thirteen male amateur soccer players from the same team, who competed at regional level, participated in this study (age: 27.07 ± 4.11 years; height: 176.33 ± 5.14 cm; body mass: 72.61 ± 7.62 kg). The inclusion criteria for the sample were as follows: participants had no medical conditions or acute injuries during the data collection period, and they trained three times (~90 min per session) the week before the data collection period. Goalkeepers were excluded from the study because their match-related demands are completely different from those of outfield players.

### 2.2. Design

A repeated-measures observational design was used for this study. The data collection period took place during the competitive period (i.e., mid-season). Pre-match measurements were obtained on the morning of the match day (10:00 h). Post-match measurements were taken immediately after the match, and again at 24 h and at 48 h post-match. The official soccer match was played on a natural grass pitch against a team of the same competitive level (15:30 h). It lasted 90 min with a 15 min half-time. The team’s coaching freely decided the starting line-up and substitutions without any intervention from the research staff. Participants trained 20 h before the match as the team regularly did during the in-season period and were not involved in any type of training or physical activity during the 48 h recovery period.

As previously suggested [5,8], blood biomarkers, neuromuscular status, and perceived wellness were measured to detect neuromuscular fatigue and to monitor the time course of recovery of the participants. Post-match, 24 h, and 48 h measurements were performed at the same time of day to avoid circadian variation. All measurements were conducted in a laboratory setting with guaranteed hygienic conditions and similar environmental conditions.

Neuromuscular status was evaluated using the countermovement jump (CMJ), a suitable, non-invasive test for use in athlete fatigue monitoring [18]. Test instructions were provided during a recruitment session two weeks before the data collection period, and participants practiced the tests during training sessions before data collection. The same standardised warm-up was performed after perceived wellness and blood biomarker data collection and before each CMJ testing session. This warm-up was selected based on previous suggestions that a general and specific warm-up (dynamic active stretching) leads to superior gains in CMJ performance [19]. Accordingly, participants performed five minutes running and dynamic active stretching, which consisted of seven exercises performed in seven minutes. Exercises were straight leg march, butt kicks, carioca, high knees, reverse lunge with twist, power shuffle (step slide), and jogging with squats. Each exercise consisted of two 20 s sets with a 10 s rest interval between sets and exercises.

Participants were required to refrain from taking anti-inflammatory drugs, nutritional or multi-vitamin supplements, antioxidants, or other prescription drugs for seven days before the study and throughout the recovery period. They were also advised to abstain from consuming alcohol or caffeine 24 h before the pre-match measurements and during the data collection period. During the match, participants could drink only water.

### 2.3. Blood Biomarkers

Blood collection and sample management were performed in strict accordance with guidelines for improving the preanalytical quality of sports biochemistry and haematology tests [20]. After a five-hour fast, blood samples were collected from an antecubital arm vein into serum separator tubes (SST^TM^ II advance, BD Vacutainer^®^, Becton Dickinson, San Agustin de Guadalix, Spain). Two 5 mL tubes of blood were obtained. Blood samples were allowed to clot at room temperature and plasma and serum were subsequently separated by centrifugation (3500 rpm, 4 ± 0.5 °C) using an automated centrifuge (Inilab 8, Inilab, Arganda del Rey, Spain).

The following biomarkers were monitored for different purposes [11,21,22]: creatine kinase (CK; U·L^−1^) and lactate dehydrogenase (LDH; U·L^−1^) to detect EIMD, C-reactive protein (CRP; mg·L^−1^) to assess inflammatory response, uric acid (UA; mg·dL^−1^) to evaluate oxidative stress, and testosterone (T; ng·mL^−1^), cortisol (C; µg·dL^−1^), and testosterone to cortisol ratio (T:C ratio) to determine endocrine response and anabolic/catabolic status. Using a clinical chemistry and turbidimetry analyser (BioSystems BA200, BioSystems, Barcelona, Spain), CK and LDH levels were determined by measuring absorbance at 340 nm, CRP concentration was obtained using the turbidimetric method, and UA level was determined spectrophotometrically. T and C concentrations were measured using immunoreactivity-activated procedures (chemiluminescence) and an automated immunoassay analyser (Alinity I System, Abbott Laboratories, Abbott Park, IL, USA).

### 2.4. Neuromuscular Status

Neuromuscular responses to match-play were measured using CMJ performance. Participants began from an upright position with straight legs and hands on their hips to eliminate the contribution of arm swing to jump height. Once in the starting position, they remained as still as possible for at least three seconds to allow for the collection of their body weight. Participants executed a downward movement before the jump, performing a natural flexion before take off, and were asked to land in an upright position while bending their knees. The CMJ depth was self-selected by the participant to avoid any alterations in their preferred jump strategy. All CMJ trials were recorded using a Kistler Quattro Jump type 9290DD force plate with MARS for Quattro Jump & KiJump 5.2 software (Kistler Group, Winterthur, Switzerland), sampling at 500 Hz. Each participant performed two CMJ trials with two minutes of recovery between them. The average value of both trials was included in the statistical analysis, as it has been reported to be more sensitive than the highest CMJ value to monitor changes in neuromuscular status [23].

CMJ-derived metrics were selected considering that some output metrics may be less sensitive than other time-related metrics in detecting neuromuscular fatigue or measuring recovery status [24,25]. In fact, athletes with fatigue-induced changes in neuromuscular function may adjust their jump strategy to produce the same force and achieve the same jump height [18,24]. Thus, the following metrics were selected to describe both outcome and movement strategy: jump height (vertical displacement of the participant’s centre of mass; cm), absolute peak power (AbsPeakP; highest power during the jump; W), relative peak power (RelPeakP; highest power relative to the participant’s body mass during the jump; W·kg^−1^), propulsive impulse (PropImp; product of force and time during ascent; Ns), peak rate of force development (PeakRFD; greatest amount of force in a given amount of time; N·s^−1^), time to take off (TTakeOff; total duration from jump initiation -start of movement- to take off; s), propulsive phase duration (PropPhDur; time spent during ascent prior to take off; s), time to peak force (TPeakF; amount of time it takes before the greatest amount of force is produced; s), reactive strength index modified (RSImod; ratio of jump height to contraction time or time spent from jump initiation -start of movement- to take off) and flight time to contraction time ratio (FT:CT; ratio of flight time to contraction time or time spent from jump initiation -start of movement- to take off).

### 2.5. Perceived Wellness

Since wellness is a multidimensional construct [26], it was monitored using the Hooper questionnaire [27]. A seven-point Likert scale (scores 1–7) was used to assess participants perceived fatigue, DOMS, sleep quality, and stress in arbitrary units (au). The verbal anchors for fatigue, DOMS and stress levels were “very very low” (1) and “very very high” (7), while for sleep quality they were “very very good” (1) and “very very bad” (7). The questionnaire was administered individually to minimise factors that might influence participants’ wellness rating, such as peer pressure or replicating other participants’ scores. Participants were already familiar with this psychometric tool, as they completed it daily as a part of their team’s monitoring routine after the recruitment session.

### 2.6. Statistical Analyses

Descriptive statistics are reported as means and standard deviations (M ± SD). The intraclass correlation coefficient (ICC) was used to determine the inter-session reliability for each measurement [28]. ICC values were interpreted using the following scale [29]: poor (<0.5), moderate (0.5–0.75), good (0.75–0.9), and excellent (>0.9). The Shapiro–Wilk test was applied to evaluate the normal distribution of the data, and Levene’s test was used to assess the homogeneity of variances. Differences between time-points were compared using both parametric and nonparametric tests (i.e., one-way repeated measures analysis of variance—ANOVA—with a Holm post hoc adjustment and Friedman test, respectively). Differences between time-points were also compared using standardised differences based upon Cohen’s effect size (ES) and qualitative probabilistic inference with 90% confidence intervals. Inferences were based on standardised thresholds for the smallest worthwhile change, which was set as 0.2 of baseline SD [30]. Standardised differences were considered trivial (<0.2), small (0.2–0.5), moderate (0.5–0.8), or large (>0.8) [31]. The qualitative probabilistic terms were most unlikely (<0.5%), very unlikely (0.5–5%), unlikely (5–25%), possibly (25–75%), likely (75–95%), very likely (95–99.5%), or most likely (>99.5%) [32]. Statistical significance was established at *p* < 0.05. Data analysis was performed using JASP 0.16.3.0 software (University of Amsterdam, Amsterdam, The Netherlands).

## 3. Results

Participants played for an average of 79.62 ± 22.05 min. Three participants were substituted during the second half, while seven played the full match (90 min).

The following variables reported a poor ICC: UA, C, T:C ratio, jump height, PeakRFD, TTakeOff, TPeakF, RSImod, FT:CT, fatigue, DOMS, sleep quality, and stress (0.406, 0.112, 0.215, 0.440, 0.492, 0.277, 0.286, 0.218, 0.226, 0.297, 0.244, 0.107, and 0.255, respectively). CK, LDH, CRP, T, RelPeakP, PropImp, and PropPhDur reported a moderate ICC (0.520, 0.642, 0.637, 0.535, 0.725, 0.651, and 0.566, respectively). Only AbsPeakP reported a good ICC (0.884).

**Figure 1:**
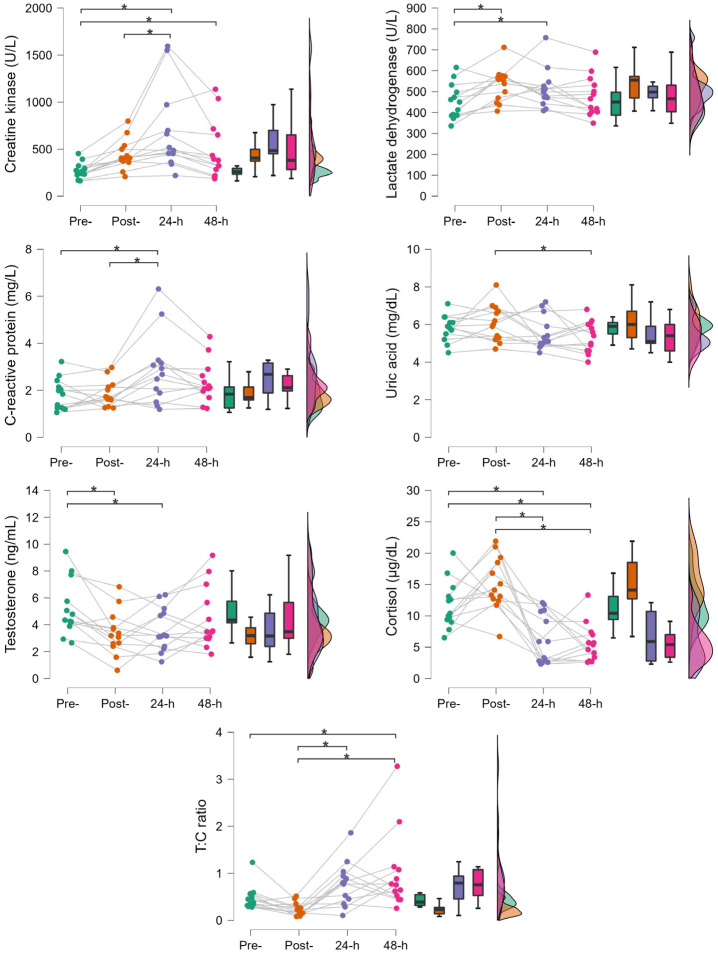
Time course changes in biomarkers of exercise-induced muscle damage (creatine kinase, lactate dehydrogenase), inflammation (C-reactive protein), oxidative stress (uric acid), and endocrine response (testosterone, cortisol, testosterone to cortisol ratio). Each panel includes a sinaplot with individual changes and mean differences (* *p* < 0.05), a box plot with median values and the interquartile range, and a half-violin plot with the kernel probability density of the values.

Statistical significance was found for EIMD and inflammatory biomarkers (Figure 1). CK levels peaked 24 h post-match, where they were significantly higher than pre- (273.46 ± 81.09 vs. 675.62 ± 439.71; *p* < 0.001; ES 1.42, large; most likely) and post-match (441.00 ± 158.51 vs. 675.62 ± 439.71; *p* = 0.022; ES 0.83, large; very likely) levels. CK levels remained significantly elevated at 48 h compared to pre-match (273.46 ± 81.09 vs. 483.85 ± 311.42; *p* = 0.039; ES 0.74, moderate; very likely) levels. LDH peaked at post-match, where it was significantly higher than pre-match levels (452.85 ± 83.82 vs. 532.31 ± 80.55; *p* = 0.003; ES 0.90, large; very likely), and remained increased at 24 h post-match (452.85 ± 83.82 vs. 512.31 ± 91.97; *p* = 0.035; ES 0.67, moderate; likely). The CRP peak was at 24 h after the match, where it was significantly higher than pre- (1.82 ± 0.67 vs. 2.86 ± 1.49; *p* < 0.001; ES 1.08, large; most likely) and post-match levels (1.87 ± 0.55 vs. 2.86 ± 1.49; *p* < 0.001; ES 1.03, large; most likely), but it returned to baseline at 48 h post-match.

Regarding oxidative stress (Figure 1), a significant response was also found for UA, but it was only significantly reduced at 48 h post- compared to post-match (6.08 ± 0.97 vs. 5.34 ± 0.83; *p* = 0.040; ES −0.87, large; very likely). There were no significant differences among other time-points.

Endocrine responses also showed significant differences (Figure 1). T levels were significantly decreased at both post- (5.17 ± 2.04 vs. 3.39 ± 1.64; *p* < 0.008; ES −0.93, large; very likely) and 24 h post-match (5.17 ± 2.04 vs. 3.65 ± 1.61; *p* < 0.026; ES −0.79, moderate; very likely) before returning to baseline at 48 h post-match. C levels did not show significant differences between pre- and post-match, but post-match values were significantly higher than those at 24 h (15.12 ± 4.25 vs. 6.39 ± 3.96; *p* < 0.001; ES −2.32, large; most likely) and 48 h post-match (15.12 ± 4.25 vs. 5.68 ± 3.03; *p* < 0.001; ES −2.50, large; most likely). In fact, compared to baseline, C levels were significantly lower at 24 h (11.72 ± 3.75 vs. 6.39 ± 3.96; *p* = 0.003; ES −1.41, large; most likely) and 48 h post-match (11.72 ± 3.75 vs. 5.68 ± 3.03; *p* < 0.001; ES −1.60, large; most likely). Despite no significant differences between pre- and post-match, the lowest T:C ratio was measured post-match, where it was significantly lower than the values at 24 h (0.24 ± 0.14 vs. 0.78 ± 0.46; *p* = 0.017; ES 1.09, large; very likely) and 48 h post-match (0.24 ± 0.14 vs. 1.00 ± 0.83; *p* < 0.001; ES 1.53, large; most likely). The T:C ratio at 48 h post-match was significantly higher than baseline (0.47 ± 0.25 vs. 1.00 ± 0.83; *p* = 0.018; ES 1.06, large; very likely).

Results from the CMJ-derived metrices indicate that neuromuscular performance was significantly impaired both after the match and during the recovery period (Table 1). Jump height was significantly decreased at 24 h (*p* = 0.007) and 48 h post-match (*p* = 0.045), but not immediately after the match. PeakRFD was significantly reduced immediately after the match (*p* = 0.018) and remained reduced at 24 h (*p* = 0.018) before returning to baseline at 48 h post-match. TTakeOff was significantly increased immediately after the match compared to pre- (*p* = 0.001) and 48 h post-match (*p* = 0.017). PropPhDur was significantly increased at 24 h post-match compared to baseline (*p* = 0.016). TPeakF was significantly increased immediately after the match compared to pre- (*p* < 0.001) and 48 h post-match (*p* = 0.014). RSImod was significantly reduced at post- compared to baseline (*p* = 0.049). Similarly, FT:CT was significantly decreased at post- compared to pre-match (*p* = 0.028). No significant differences between time-points were found for AbsPeakP, RelPeakP, and PropImp.

Some significant differences between time-points were found for perceived wellness (Table 2). Perceived fatigue peaked immediately after the match, where it was significantly higher than pre- (*p* < 0.001) and 48 h post-match (*p* < 0.001). It remained significantly elevated at 24 h (*p* < 0.001) and then significantly decreased at 48 h post-match (*p* = 0.014). DOMS was significantly increased at post-match (*p* = 0.002), peaked at 24 h (*p* < 0.001), and returned to baseline 48 h after the match. No significant differences between time-points were found for sleep quality and stress.

## 4. Discussion

This study analysed the recovery process after an official soccer match by monitoring changes in markers of muscle damage and oxidative stress, and endocrine, neuromuscular, and perceptual responses. Except for CK, which remained elevated at 48 h post-match, the other blood biomarkers returned to baseline between 24 h and 48 h post-match. Jump height was significantly decreased at 24 h and 48 h post-match, while PeakRFD and other CMJ time-based metrics (i.e., TTakeOff, TPeakF, RSImod, FT:CT) were impaired immediately after the match and recovered earlier. Peak values for perceived fatigue and DOMS were observed immediately post- and at 24 h post-match, respectively. These findings highlight the importance of considering multiple physiological, neuromuscular, and perceptual responses to soccer match-loads [1,5,8].

The high demands of eccentric muscle contractions characteristic of soccer-related movements (i.e., changes of direction, decelerations, backward and sideways running, jumps, and tackles) may expose players to mechanical strain and subsequent EIMD, leading to structural disruptions of the contractile elements within muscle fibres [9]. As a result of structural disruptions from EIMD, CK and LDH are released into the systemic circulation [10,11,21]. These structural disarrangements are also followed by an inflammatory response, indicated by increased levels of CRP [10,11,21]. The current results indicate that the soccer match-play induced EIMD with a subsequent inflammatory response. While LDH peaked at post-match, CK and CRP were not significantly increased at this time-point and instead peaked at 24 h post-match. LDH and CRP returned to baseline at 48 h post-match, but CK remained increased at this time-point. The time course recovery of these biomarkers was relatively similar to what is reported in previous studies. Specifically, CK and LDH levels tend to remain elevated for 48 h to 72 h after soccer matches, tough LDH levels may peak and recover earlier than CK, and CRP concentrations typically peak at 24 h post-match with baseline values being restored within 72 h [6,7,11].

Prolonged and damaging soccer-related movements can lead to oxidative stress, which in turn contributes to the acute-phase inflammatory response from EIMD and has been linked to reduced muscle force production [14]. Oxidative stress is indicated by changes in circulating levels of oxidated and antioxidative molecules, which can remain elevated for days after a soccer match [6,11]. UA levels have been shown to directly correlate with total antioxidant capacity [14] and are frequently used to report increased purine metabolism and compensatory changes in antioxidant capacity in response to soccer match-play. In the current study, UA decreased at 48 h post- compared to post-match, with no significant differences found between other time-points. This result is consistent with previous findings that indicate plasma UA levels tend to increase immediately after a soccer match and can remain elevated at 48 h post-match [6,7,11]. The absence of differences between pre- and post-match values may be explained by elevated resting levels resulting from the training that participants performed 20 h before blood sampling [22]. This preanalytical condition (i.e., refraining from intense exercise before blood sampling) was impossible to fulfil during the data collection period.

Previous evidence indicates that soccer matches induce immediate endocrine responses, with decreased T and increased C concentrations [6,11,12]. T levels during recovery may not be altered [12], but if a decrease occurred, it may persist until 48 h post-match [6]. The current results support these findings, as T concentrations were significantly decreased both post- and at 24 h post-match before returning to baseline at 48 h. C levels typically remain elevated for the following 24 h and 48 h [6,11,12], or even up to 72 h post-match [15]. However, the current study found no significant differences in C or T:C ratio between pre- and post-match. In fact, C concentrations were significantly lower at 24 h and 48 h post-match compared to pre- and post-match values, while the T:C ratio was significantly lower at pre-match compared to 48 h post-match, and also immediately after the match compared to 24 h and 48 h post-match. These findings may be explained by the elevated C resting levels resulting from the training performed 20 h before blood sampling [22]. Additionally, the large individual variability in endocrine responses can be influenced by psycho-physiological stress (i.e., changes in cognitive anxiety), match outcome (i.e., win, draw, loss), and the type of competition (i.e., official, friendly) [12].

The current results indicate that neuromuscular performance was significantly impaired both after the match and during the recovery period. This has been widely supported by previous research, as a soccer match often leads to substantial muscle function impairments that can last for 48 h to 72 h, with alterations in muscle contractile properties and central motor output [6,7,8]. However, a novel aspect of this study is the inclusion of several CMJ-derived metrics not typically considered in prior research. A previous meta-analysis reported that jump height and peak power were the most analysed metrics for detecting CMJ neuromuscular fatigue [23]. However, using output metrics such as jump height, AbsPeakP, RelPeakP, PropImp, or PeakRFD may limit their applicability for neuromuscular fatigue monitoring, as they appear to be adequate for neuromuscular performance profiling but may be less sensitive to detecting neuromuscular fatigue than other CMJ-derived metrics [25]. These limitations have been recently confirmed in youth elite soccer players [33]. In contrast, time-based metrics (i.e., TTakeOff, PropPhDur, TPeakF, RSImod, FT:CT) may be more appropriate for detecting neuromuscular fatigue or measuring recovery status [24,25]. In fact, athletes with fatigue-induced changes in neuromuscular function may adjust their jump mechanics to produce the same force and maintain jump height [18,24]. The current study confirmed these discrepancies between alterations in jump strategy and neuromuscular function, as jump height was not reduced immediately after the match while PeakRFD and other time-based metrics (i.e., TTakeOff, TPeakF, RSImod, FT:CT) were significantly impaired at that same time-point. Furthermore, defining a single post-exercise recovery timeframe for CMJ performance is difficult, as recovery kinetics differ both within and between the CMJ-derived metrics of interest. On one hand, jump height was significantly decreased at 24 h and 48 h post-match, whereas PeakRFD was significantly reduced post- and at 24 h post-match, before returning to baseline at 48 h post-match. On the other hand, while both TTakeOff and TPeakF followed a similar time course of recovery, RSImod and FT:CT were only impaired immediately after the match. While this study cannot determine the exact mechanisms responsible for the alterations in jump strategy and neuromuscular function after a soccer match, some structural and neural processes are speculated to be involved [13]. These include muscle microtrauma, metabolic disturbances, impaired excitation–contraction coupling, and a reduction in muscle stiffness related to stretch–reflex sensitivity [10,34,35]. Thus, there is a clear need for a better understanding of both the recovery kinetics of CMJ performance and the underlying neuromuscular fatigue mechanisms following a soccer match.

Regarding self-reported measures of wellness, the current results support previous findings which indicate that perceived fatigue typically peaks at match-end and DOMS after 24 h of recovery, and both can remain substantially elevated for up to 72 h post-match [6]. DOMS is a transient phenomenon that follows EIMD and is linked to inflammatory responses, systemic release of myocellular enzymes and proteins, loss of muscle force and power, and reduced range of motion [10,36]. It may manifest when the injured non-nociceptive sensory fibres of the muscle spindle stop inhibiting the effects of the injured, hyperexcited nociceptive sensory fibres [37]. Therefore, the analogous responses between perceived wellness (i.e., fatigue, DOMS) and objective measures of EIMD (i.e., CK, LDH), inflammation (i.e., CRP), and decreased performance (i.e., CMJ-derived metrics) support, at least in part, the relationships between physiological and psychological time courses of recovery after soccer matches.

The following limitations should be noted. First, the poor to moderate reliability of most metrics could influence the current results by introducing a higher degree of random error, which may lead to an underestimation of the true effects. Second, due to the great individual and match-to-match variability shown in the literature [4,5,8,12,23], the results may also be influenced by the small sample and number of observations. Accordingly, further research is encouraged in official matches including larger samples and a wider range of age categories and sex to enhance the generalizability of these findings. Third, blood biomarkers, neuromuscular status, and perceived wellness were not measured at 72 h post-match. Therefore, future research should incorporate a 72 h or even longer measurement to fully describe the complete time course recovery and better inform training and competition schedules. Finally, equipment availability did not allow authors to measure the players’ external loads during the official match, which may have provided a real quantification of mechanical stress. Thus, the analysis of match running performance is also recommended to better explain the physiological and psychological responses during the recovery period, which is essential for establishing clear cause-and-effect relationships.

## 5. Conclusions

The results of the current study indicate that an official amateur-level soccer match imposes considerable stress on players, as shown by several physiological, neuromuscular, and perceptual responses that can persist for days. However, blood biomarkers, CMJ-derived metrics of neuromuscular status, and perceived wellness exhibited divergent responses immediately after the match and required different recovery periods for complete restoration. In fact, while certain physiological, neuromuscular, and perceptual changes may return to baseline levels within 24 h or 48 h post-match, amateur soccer players still manifest EIMD symptoms and can be considered fatigued after a 48 h recovery period. These results may provide valuable insights for practitioners to guide training schedules and recovery protocols in real-world conditions.

## Figures and Tables

**Table 1:** Mean differences, standardised differences, and qualitative probabilistic inference of CMJ-derived metrics between time-points.

Metric	Pre-Match	Post-Match	24 h Post-Match	48 h Post-Match	Comparison	*p*	ES, Magnitude	Qualitative Inference
**Height** **(cm)**	41.29 ± 7.56	37.80 ± 3.15	36.13 ± 4.21	37.23 ± 4.90	Pre–post	0.093	−0.67 (moderate)	Likely
					Pre–24 h	0.007 *	−0.99 (large)	Very likely
					Pre–48 h	0.045 *	−0.78 (moderate)	Likely
					Post–24 h	0.788	−0.32 (small)	Possibly
					Post–48 h	0.917	−0.11 (trivial)	Possibly
					24 h–48 h	0.917	0.21 (small)	Possibly
**AbsPeakP** **(W)**	4107.18 ± 636.53	3866.68 ± 645.25	4069.29 ± 741.08	4030.54 ± 737.37	Pre–post	0.062	−0.35 (small)	Likely
					Pre–24 h	1.000	−0.06 (trivial)	Most unlikely
					Pre–48 h	1.000	−0.11 (trivial)	Most unlikely
					Post–24 h	0.144	0.29 (small)	Possibly
					Post–48 h	0.295	0.24 (small)	Possibly
					24 h–48 h	1.000	−0.06 (trivial)	Most unlikely
**RelPeakP** **(W·kg^−1^)**	55.61 ± 5.99	52.92 ± 5.59	54.49 ± 5.65	53.74 ± 6.03	Pre–post	0.149	−0.46 (small)	Likely
					Pre–24 h	1.000	−0.19 (trivial)	Most unlikely
					Pre–48 h	0.562	−0.32 (small)	Possibly
					Post–24 h	0.721	0.27 (small)	Possibly
					Post–48 h	1.000	0.14 (trivial)	Most unlikely
					24 h–48 h	1.000	−0.13 (trivial)	Most unlikely
**PropImp** **(Ns)**	217.53 ± 53.01	196.40 ± 22.31	196.69 ± 31.15	201.03 ± 28.35	Pre–post	0.068	−0.59 (moderate)	Likely
					Pre–24 h	0.068	−0.58 (moderate)	Likely
					Pre–48 h	0.179	−0.46 (small)	Possibly
					Post–24 h	1.000	0.01 (trivial)	Most unlikely
					Post–48 h	1.000	0.13 (trivial)	Most unlikely
					24 h–48 h	1.000	0.12 (trivial)	Most unlikely
**PeakRFD** **(N·s^−1^)**	15,059.54 ± 9043.17	9704.11 ± 4789.14	9781.821 ± 3449.47	12,897.61 ± 6463.72	Pre–post	0.018 *	−0.85 (large)	Very likely
					Pre–24 h	0.018 *	−0.84 (large)	Very likely
					Pre–48 h	0.420	−0.34 (small)	Possibly
					Post–24 h	0.964	0.01 (trivial)	Unlikely
					Post–48 h	0.268	0.51 (moderate)	Possibly
					24 h–48 h	0.268	0.49 (small)	Possibly
**TTakeOff** **(s)**	0.82 ± 0.13	1.01 ± 0.18	0.90 ± 0.09	0.86 ± 0.18	Pre–post	0.001 *	1.30 (large)	Most likely
					Pre–24 h	0.298	0.54 (moderate)	Likely
					Pre–48 h	0.730	0.30 (small)	Possibly
					Post–24 h	0.097	−0.75 (moderate)	Likely
					Post–48 h	0.017 *	−1.00 (large)	Very likely
					24 h–48 h	0.730	−0.25 (small)	Possibly
**PropPhDur** **(s)**	0.27 ± 0.04	0.29 ± 0.05	0.31 ± 0.06	0.29 ± 0.05	Pre–post	0.455	0.43 (small)	Possibly
					Pre–24 h	0.016 *	0.80 (large)	Very likely
					Pre–48 h	0.455	0.39 (small)	Possibly
					Post–24 h	0.455	0.37 (small)	Possibly
					Post–48 h	0.865	−0.04 (trivial)	Possibly
					24 h–48 h	0.455	−0.41 (small)	Possibly
**TPeakF** **(s)**	0.58 ± 0.14	0.80 ± 0.18	0.69 ± 0.11	0.63 ± 0.19	Pre–post	<0.001 *	1.37 (large)	Most likely
					Pre–24 h	0.145	0.69 (moderate)	Likely
					Pre–48 h	0.553	0.35 (small)	Possibly
					Post–24 h	0.145	−0.68 (moderate)	Likely
					Post–48 h	0.014 *	−1.02 (large)	Very likely
					24 h–48 h	0.553	−0.34 (small)	Possibly
**RSImod**	0.51 ± 0.16	0.39 ± 0.10	0.42 ± 0.07	0.46 ± 0.15	Pre–post	0.049 *	−0.93 (large)	Likely
					Pre–24 h	0.177	−0.73 (moderate)	Likely
					Pre–48 h	0.651	−0.42 (small)	Possibly
					Post–24 h	0.723	0.20 (small)	Possibly
					Post–48 h	0.533	0.51 (moderate)	Possibly
					24 h–48 h	0.723	0.31 (small)	Possibly
**FT:CT**	0.70 ± 0.12	0.57 ± 0.13	0.62 ± 0.07	0.67 ± 0.15	Pre–post	0.028 *	−0.99 (large)	Very likely
					Pre–24 h	0.285	−0.62 (moderate)	Likely
					Pre–48 h	0.753	−0.23 (small)	Possibly
					Post–24 h	0.753	0.38 (small)	Possibly
					Post–48 h	0.130	0.77 (moderate)	Likely
					24 h–48 h	0.753	0.39 (small)	Possibly

AbsPeakP: absolute peak power; ES: effect size; FT:CT: flight time to contraction time ratio; PeakRFD: peak rate of force development; PropImp: propulsive impulse; PropPhDur: propulsive phase duration; RelPeakP: relative peak power; RSImod: reactive strength index modified; TPeakF: time to peak force; TTakeOff: time to take off. * Statistically significant difference (*p* < 0.05).

**Table 2:** Mean differences, standardised differences, and qualitative probabilistic inference of perceived wellness between time-points.

Metric	Pre-Match	Post-Match	24 h Post-Match	48 h Post-Match	Comparison	*p*	ES, Magnitude	Qualitative Inference
**Fatigue** **(au)**	2.20 ± 0.68	4.40 ± 1.55	3.93 ± 1.34	2.80 ± 1.21	Pre–post	<0.001 *	1.78 (large)	Most likely
					Pre–24 h	<0.001 *	1.40 (large)	Most likely
					Pre–48 h	0.240	0.49 (small)	Likely
					Post–24 h	0.240	−0.38 (small)	Likely
					Post–48 h	<0.001 *	−1.30 (large)	Most likely
					24 h–48 h	0.014 *	−0.92 (large)	Very likely
**DOMS** **(au)**	2.40 ± 1.18	3.93 ± 1.44	4.00 ± 1.31	3.00 ± 1.07	Pre–post	0.002 *	1.22 (large)	Most likely
					Pre–24 h	0.001 *	1.27 (large)	Most likely
					Pre–48 h	0.281	0.48 (small)	Possibly
					Post–24 h	0.868	0.05 (trivial)	Possibly
					Post–48 h	0.073	−0.74 (moderate)	Likely
					24 h–48 h	0.065	−0.79 (moderate)	Likely
**Sleep** **(au)**	2.53 ± 1.13	NM	3.20 ± 1.61	2.47 ± 1.36	Pre–24 h	0.683	0.52 (moderate)	Possibly
					Pre–48 h	1.000	−0.02 (trivial)	Most unlikely
					24 h–48 h	0.664	−0.57 (moderate)	Possibly
**Stress** **(au)**	2.20 ± 0.94	2.67 ± 1.48	2.07 ± 0.80	2.47 ± 1.25	Pre–post	0.844	0.41 (small)	Possibly
					Pre–24 h	1.000	−0.12 (trivial)	Most unlikely
					Pre–48 h	1.000	0.24 (small)	Most unlikely
					Post–24 h	0.474	−0.53 (moderate)	Possibly
					Post–48 h	1.000	−0.18 (trivial)	Most unlikely
					24 h–48 h	0.947	0.35 (small)	Possibly

au: arbitrary unit; ES: effect size; DOMS: delayed onset muscle soreness; NM: not measured. * Statistically significant difference (*p* < 0.05).

## Data Availability

The datasets generated during and/or analysed during the current study are not publicly available due to the sensitive nature of the research supporting data. The datasets generated during and/or analysed during the current study are available from the corresponding author on reasonable request.

## Abbreviations

The following abbreviations are used in this manuscript:

AbsPeakP	Absolute peak power
C	Cortisol
CK	Creatine kinase
CMJ	Countermovement jump
CRP	C-reactive protein
DOMS	Delayed onset muscle soreness
EIMD	Exercise-induced muscle damage
ES	Effect size
FT:CT	Flight time to contraction time ratio
ICC	Intraclass correlation coefficient
LDH	Lactate dehydrogenase
PeakRFD	Peak rate of force development
PropImp	Propulsive impulse
PropPhDur	Propulsive phase duration
RelPeakP	Relative peak power
RSImod	Reactive strength index modified
T	Testosterone
TPeakF	Time to peak force
TTakeOff	Time to take off
T:C ratio	Testosterone to cortisol ratio
UA	Uric acid
